# The correlation between antimutagenic activity and total phenolic content of extracts of 31 plant species with high antioxidant activity

**DOI:** 10.1186/s12906-016-1437-x

**Published:** 2016-11-29

**Authors:** Tshepiso Jan Makhafola, Esameldin Elzein Elgorashi, Lyndy Joy McGaw, Luc Verschaeve, Jacobus Nicolaas Eloff

**Affiliations:** 1 Department of Paraclinical Sciences, Phytomedicine Programme, Faculty of Veterinary Science, University of Pretoria, Private Bag X04, Onderstepoort, 0110 South Africa; 2 Toxicology and Ethnoveterinary Medicine, Food, Feed and Veterinary Public Health, ARC-Onderstepoort Veterinary Institute, Private Bag X05, Onderstepoort, 0110 South Africa; 3 Department of Paraclinical Sciences, Faculty of Veterinary Science, University of Pretoria, Private Bag X04, Onderstepoort, 0110 South Africa; 4 Scientific Institute of Public Health, Rue Juliette Wytsmanstreet 14, 1050 Brussels, Belgium; 5 Department of Biomedical Sciences, University of Antwerp, Universiteitsplein 1, B-2610 Wilrijk, Belgium

**Keywords:** Antimutagenicity, Ames test, Antioxidant activity, Total phenolic content, Plant extracts

## Abstract

**Background:**

Antimutagenic activity of plant extracts is important in the discovery of new, effective cancer preventing agents. There is increasing evidence that cancer and other mutation-related diseases can be prevented by intake of DNA protective agents. The identification of antimutagenic agents present in plants presents an effective strategy to inhibit pathogenic processes resulting from exposure to mutagenic and/or carcinogenic substances present in the environment. There are no reports on the antimutagenic activities of the plant species investigated in this study. Many mutations related to oxidative stress and DNA damage by reactive oxygen species (ROS) and reactive nitrogen species (RNS) have been identified in numerous human syndromes. Oxidative DNA damage plays a significant role in mutagenesis, cancer, aging and other human pathologies. Since oxidative DNA damage plays a role in the pathogenesis of several chronic degenerative diseases, the decrease of the oxidative stress could be the best possible strategy for prevention of these diseases. Antioxidant compounds can play a preventative role against mutation-related diseases, and thus have potential antimutagenic effects.

**Methods:**

The number of antioxidant compounds present in methanol leaf extracts of 120 plant species was determined using a combination of Thin Layer Chromatography (TLC) and spraying with 2, 2-diphenyl-1-picrylhydrazyl (DPPH). The 31 most promising extracts were selected for further assays. The quantitative antioxidant activity was determined using DPPH free radical scavenging spectrophotometric assay. Total phenolic contents were determined using the Folin-Ciocalteu colorimetric assay. The mutagenicity of 31 selected extracts was determined in the Ames test using *Salmonella typhimurium* strains TA98 and TA100. The antimutagenicity of the plant extracts against 4-nitroquinoline 1-oxide (4-NQO) was also determined using the Ames test.

**Results:**

Of the 120 plant extracts assayed qualitatively, 117 had some antioxidant activity. The selected 31 extracts contained well defined antioxidant compounds. These species had good DPPH free radical antioxidant activity with EC_50_ values ranging from 1.20 to 19.06 μg/ml. Some of the plant extracts had higher antioxidant activity than L-ascorbic acid (vitamin C). The total phenolic contents ranged from 5.17 to 18.65 mg GAE (gallic acid equivalent)/g plant extract). The total phenolic content of the plant extracts correlated well with the respective antioxidant activity of the plant extracts. No plant extract with good antioxidant activity had mutagenic activity. Several extracts had antimutagenic activity. The percentage inhibition of 4-NQO ranged from 0.8 to 77% in *Salmonella typhimurium* TA98 and from 0.8 to 99% in strain TA100. There was a direct correlation between the presence of antioxidant activity and antimutagenic activity of the plant extracts. Although no plant extract had mutagenic activity on its own, some of the plant extracts enhanced the mutagenicity of 4-NQO, a phenomenon referred to as comutagenicity.

**Conclusions:**

Some of the plant extracts investigated in this study had potential antimutagenic activities. The antimutagenic activities may be associated with the presence of antioxidant polyphenols in the extracts. From the results plant extracts were identified that were not mutagenic, not cytotoxic and that may be antimutagenic in the Ames test. For most plant extracts, at the highest concentration used (5 mg/ml), the level of antimutagenicity was below the recommended 45% to conclude whether plants have good antimutagenic activity. However, in most screening studies for antimutagenesis, a 20% decrease in the number of revertants must be obtained in order to score the extract as active. *Psoralea pinnata* L. had the highest percentage antimutagenicity recorded in this study (76.67 and 99.83% in *S. typhimurium* TA98 and TA100 respectively) at assayed concentration of 5 mg/ml.

The results indicate that investigating antioxidant activity and the number of antioxidant compounds in plant extracts could be a viable option in searching for antimutagenic compounds in plants.

**Electronic supplementary material:**

The online version of this article (doi:10.1186/s12906-016-1437-x) contains supplementary material, which is available to authorized users.

## Background

Plants have formed the basis of many traditional medicine systems throughout the world for centuries and continue to provide mankind with new remedies. Numerous useful drugs have been developed from lead compounds discovered from medicinal plants. Up to this day, this strategy remains an important route to new pharmaceuticals [1]. Medicinal plants are important in health care systems of developing countries for primary health care needs. The popularity of medicinal plants is connected with their easy access, claimed therapeutic efficacy based on local knowledge and expertise amongst the local communities as well as affordability [2]. Plants contain many metabolites with various bioactivities including antioxidant, anti-inflammatory and anticancer activities [3]. Many bioactive compounds from plants are antioxidants and have been shown to have possible health effects, partly due to their antioxidative properties [4].

Many mutagens and carcinogens act by generating reactive oxygen species (ROS) and ROS are widely recognised for playing a harmful role in living systems by inducing oxidative damage to cell structures and biomolecules such as lipids, nucleic acids and proteins [5]. DNA mutation is a crucial step in carcinogenesis, and elevated levels of oxidative DNA lesions have been noted in many tumours, strongly implicating such damage in the aetiology of cancer. Oxidative DNA base lesions are mutagenic, thus the prevention of oxidative DNA lesions is important to limit mutagenesis, cytostasis, and cytotoxicity and may contribute to prevention of mutation-related diseases [6, 7]. For this study, 4-nitroquinoline 1-oxide (4-NQO) was the mutagen of choice. 4-NQO is a quinoline derivative and tumorigenic compound used in the assessment of the efficacy of drugs and procedures in the prevention of cancer as it produces all stages of carcinogenesis [8, 9]. 4-NQO induces potential intracellular oxidative stress [10]. It can undergo redox cycling and generates reactive oxygen species such as the superoxide radical and hydrogen peroxide [11].

The mechanisms and the types of active compounds involved in the protective effects of plants against mutations have not been clearly identified. A common factor in the pathogenesis of chronic degenerative diseases is the involvement of oxidative stress. Plant compounds may reduce oxidative stress, thereby reducing the risk of diseases [12]. To minimize the detrimental genotoxic effects of mutagens caused by exposure to free radicals, chemical compounds, air pollutants or metabolic processes, the use of natural antimutagens is a good alternative. Antimutagens that complement DNA repair systems and those that have antioxidant properties may be found in plants [13]. It is certainly worth investigating what place these compounds have in the prevention of mutations.

Recently the role of phenolics in the prevention of free radical-mediated diseases has become more important. Due to their antimutagenic/anticarcinogenic activities, phenolic compounds (simple phenols, phenolic acids, naphthoquinones, xanthones, stilbenes, flavonoids, lignans, lignins and condensed tannins) have a major role in the chemoprevention of cancer [14]. In an attempt to correlate antioxidant activity and antimutagenic activity, we investigated the potential antimutagenic effects of 31 plant extracts with good antioxidant activities and high total phenolic content. These plant species may have applications as probes for development of antimutagenic agents of natural origin. Kaur et al. [14] reviewed studies that investigated antimutagenic and anticarcinogenic potential of polyphenols and concluded that polyphenolic compounds have a major place in the chemoprotection of cancer and as a result, it is certainly worth investigating what role these compounds have in the prevention of cancer.

## Methods

### Extraction

Dried ground leaves of 120 plant species that were available were obtained from the Tree Screening Project of the Phytomedicine Programme, University of Pretoria. Voucher specimens of every plant screened were deposited at the Herbarium of the University of Pretoria [15]. A list of the species examined is attached as Additional file 1. Separate aliquots of 2 g of the powdered leaves were weighed into 50 ml polyester centrifuge tubes followed by the addition of 20 ml of methanol (technical grade, Merck chemicals). The tubes were shaken vigorously on a Labotec shaking machine for 30 min. The tubes were then centrifuged at 4000 × *g* for 15 min and the extracts were decanted into preweighed glass vials through Whatman No.1 filter paper and concentrated to dryness under a stream of cold air. After drying, the vials were reweighed to determine quantity extracted.

### Qualitative antioxidant activity (Thin Layer Chromatography)

The dried plant extracts were resuspended in methanol to a stock solution of 10 mg/ml to be used in subsequent bioassays. From the stock solution, 10 μl samples were loaded onto thin layer chromatography (TLC) plates (Merck, Kieselgel 60 F254) in a 1 cm band and developed in EMW (ethyl acetate/methanol/water = 40:5.4:4), one of the polar mobile phases developed and used in the Phytomedicine laboratory of the University of Pretoria [16]. After development, the plates were visualized under UV light and thereafter sprayed with 0.2% 2, 2-diphenyl-1-picrylhydrazyl (DPPH) in methanol reagent spray to detect antioxidant compounds [17, 18]. From the 120 plant extracts, 31 plant species with well-defined antioxidant bands were selected for further analysis.

### Quantitative antioxidant activity

The DPPH free radical scavenging spectrophotometric method described by Mensor et al. [19] and modified by Aderogba et al. [20] was used to determine the quantitative antioxidant activity. Reactions were carried out in 96-well microtitre plates and each of the crude extracts was tested at different concentrations. Blank solutions were prepared with methanol only while the negative control was DPPH solution (20 μl plus 50 μl methanol). Test sample solution (50 μl) contained plant extracts serially diluted in methanol. Methanol served as a blank for the microplate reader and the decrease in absorbance was measured at 515 nm. Percentage antioxidant activity (AA%) values were calculated from the absorbance values using the formula:

$$ \mathrm{AA}\% = 100-\left\{\left[\left(\mathrm{Abs}\ \mathrm{sample}\hbox{--} \mathrm{Abs}\ \mathrm{blank}\right)\mathrm{x}100\right]/\mathrm{Abs}\kern0.24em \mathrm{control}\right\} $$

(Abs sample is the absorbance of the sample, Abs blank is the absorbance of the blank and Abs control is the absorbance of the control). L-ascorbic acid (vitamin C) was used as a positive control (antioxidant agent). The EC_50_ value, defined as the concentration of the sample leading to 50% reduction of the initial DPPH concentration, was calculated from the separate linear regression of plots of the mean percentage of the antioxidant activity against concentration of the test extracts obtained from the three replicate assays. The results are expressed as EC_50_ values obtained from the regression plots.

### Total phenolic content

The Folin-Ciocalteu colorimetric method described by Singleton and Rossi [21] was used to determine the total phenolic content of the 31 methanol plant extracts. The Folin-Ciocalteu method uses gallic acid as a standard phenolic compound. One hundred microlitres of the 1 mg/ml extracts was mixed with 0.9 ml of distilled water and 0.1 ml Folin–Ciocalteu reagent. After 5 min, 1 ml of 7% sodium carbonate solution was added and the volume was made up to 2.5 ml with distilled water. The absorbance of the resulting blue-coloured solution was measured at 765 nm after 2 h with intermittent shaking. Quantitative measurements were performed, based on a standard calibration curve of seven points from 0.0078 to 1 mg/ml of gallic acid in methanol. The total content of phenolic compounds in the plant extracts in gallic acid equivalents (GAE) were calculated using the following formula:

$$ \mathsf{C} = \mathrm{c}.\ \mathrm{V}/\mathrm{m} $$

Where C is the total content of phenolic compounds, mg/g plant extract, in GAE; c is the concentration of gallic acid established from the calibration curve, mg/ml; V is the volume of extract, ml; and m is the mass of plant material extracted with methanol from 1 g of plant material [22].

### Ames test

The Ames test [23] was performed with *S. typhimurium* strain TA98 (which detects frame-shift mutations) and TA100 (which detects base-pair substitutions). Briefly, 100 μl of bacterial stock were incubated in 20 ml of Oxoid Nutrient broth for 16 h at 37 °C on a rotative shaker. One hundred microlitres of this overnight culture, was mixed with 2.0 ml of top agar (containing histidine-biotin) together with 0.1 ml test solution and 0.5 ml phosphate buffer. For mutagenicity screening, the test solution contained 50 μl test sample and 50 μl solvent control. For antimutagenicity screening, the test solution contained 50 μl test sample and 50 μl positive control). The top agar mixture was poured over the surface of a minimal agar plate and incubated for 48 h at 37 °C. After incubation the numbers of revertant colonies (mutants) in each plate were counted.

Antimutagenicity was expressed as percentage inhibition of mutagenicity calculated using the formula below:

$$ \%\;\mathrm{inhibition}=\left[\frac{1 - \mathrm{T}}{\mathrm{M}}\right]\times 100 $$

Where T is the number of revertants per plate in the presence of mutagen and the test solution and M is the number of revertants per plate in the positive control. All cultures were prepared in triplicate (except the solvent control where five replicas were made). Absence of toxicity was confirmed when a background layer of bacterial growth was observed, which should normally be present. The positive control, 4-nitroquinoline 1-oxide (4-NQO), was used at concentrations of 2 μg/ml (TA98) and 1 μg/ml (TA100). For all the extracts tested in the current experiments, the density of background bacterial lawn was compared to that of the negative control (after 48 h) and found to have no visible differences, indicating a lack of toxicity to the bacteria at the concentration tested [23].

## Results and discussion

The TLC-based antioxidant assay is a fast and simple technique to determine the presence of free radical scavenging compounds in crude plant extracts. Moreover, DPPH is not specific to any particular class of antioxidants, and thus provides the overall antioxidant capacity of the sample [24]. Almost all the plant extracts assayed in this study contained antioxidant compounds. Of the 120 plant species examined, 117 (97.5%) had antioxidant compounds shown as fast reacting bands with high intensity yellow colour on the TLC chromatograms The three species that did not have any antioxidant activity based on this assay were: *Maerua rosmarinoides, Baphia racemosa* and *Abutilon sonneratianum*. The different intensity of the spots suggests that the extracts contain antioxidant compounds with different activities [25]. Extracts from 31 plant species that were the most active from this initial screening were selected for further study (Table 1).

**Table 1:** Plant name, family, voucher specimen number and sample reference number of 31 plant species with good antioxidant activity

Reference number/Plant name	Family	Voucher specimen number
1. *Acalypha glabrata* Thunb.	Euphorbiaceae	PRU 1144674
2. *Dalbergia nitidula* Baker	Fabaceae	PRU 114678
3. *Halleria lucida* L*.*	Scrophulariaceae	PRU 119037
4. *Putterlickia restrospinosa* A.E. van Wyk & Mostert.	Celastraceae	PRU 114689
5. *Thespesia acutiloba* (Baker f.) Exell & Mendonça	Malvaceae	PRU 114692
6. *Alchornea hirtella* Benth	Euphorbiaceae	PRU 114699
7. *Androstachys johnsonii* Prain	Picrodendraceae	PRU 114701
8. *Argomuellera macrophylla* Pax	Euphorbiaceae	PRU 114703
9. *Brachystegia spiciformis* Benth.	Fabaceae	PRU 114705
10. *Kirkia wilmsii* Engl.	Kirkiaceae	PRE 580129
11. *Elaeodendron transvaalense* (Burtt Davy) R.H. Archer	Celastraceae	PRU 119038
12. *Cassinopsis ilicifolia* (Hochst.) Sleumer	Icacinaceae	PRU 119039
13. *Dais cotinifolia* L.	Thymelaeaceae	PRE 578648
14. *Faurea saligna* Harv.	Proteaceae	PRU 119040
15. *Harpephyllum caffrum* Bernh.	Anacardiaceae	PRU 119041
16. *Combretum microphyllum* Klotzsch	Combretaceae	LNBG 259/1995
17. *Leucospermum erubescens* Rourke	Proteaceae	PRU 119042
18. *Loxostylis alata* A. Spreng. ex. Rchb*.*	Anacardiaceae	PRE 584183
19. *Podocarpus henkellii* Stapf ex Dallim. & B.D..B. Jacks*.*	Podocarpaceae	PRE 818945
20. *Protea rubropilosa* Beard	Proteaceae	PRU 1109043
21. *Ochna gamostigmata* Du Toit	Ochnaceae	KNBG 1425/14
22. *Buxus natalensis*(Oliv.) Hutch.	Buxaceae	PRU 1109044
23. *Morella serrata* (Lam.) Killick	Myricaceae	PRU 1109045
24. *Gomphostigma virgatum* (L.f.) Baill.	Scrophulariaceae	UP 4192
25. *Ochna serullata* Walp.	Ochnaceae	UP 302
26. *Mimetes cucullatus* R. Br.	Proteaceae	PRU 1109046
27. *Protea mundii* Klotzsh	Proteaceae	PRU 1109047
28. *Protea cynaroides* (L.) L.	Proteaceae	PRU 1109048
29. *Protea neriifolia* R. Br.	Proteaceae	PRU 119049
30. *Protea nitida* Mill.	Proteaceae	PRU 119050
31. *Psoralea pinnata* L.	Leguminosae	PRU 119051

PRU = HGWJ Schweickerdt Herbarium, PRE = Pretoria National Botanical Garden, KNBG = Kirstenbosch National Botanical Garden, UP = Manie van der Schiff Botanical Garden

**Fig. 1:**
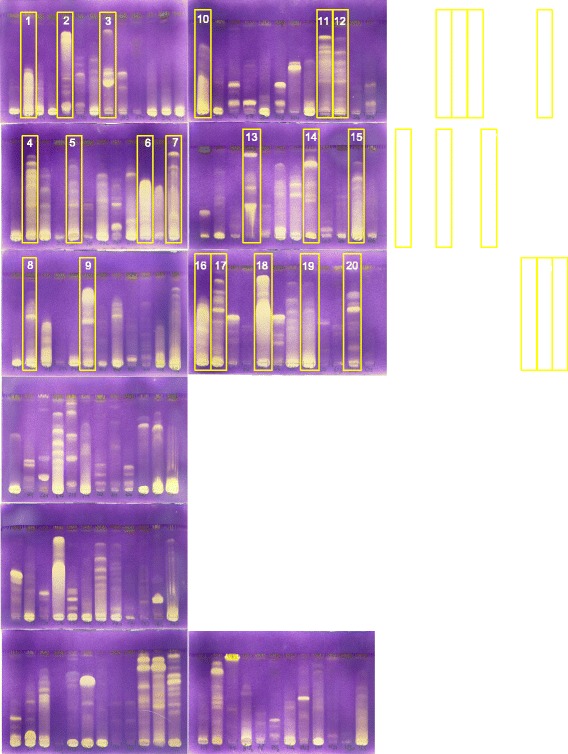
Chromatograms of the qualitative antioxidant activity of 31 out of 120 methanol plant extracts selected based on good quantitative antioxidant activity. Chromatograms were developed with ethyl acetate: methanol: water (40.5:5.4:4) sprayed with DPPH and R_f_ of compounds with antioxidant activity indicated by inhibition of colour development

All the selected 31 plant extracts effectively reduced the DPPH free radical with EC_50_ values ranging from 1.20 ± 0.22 to 19.06 ± 1.50 μg/ml (Table 2). The radical scavenging properties of the extracts indicate the antioxidant potential of the extracts. Out of 31 extracts assayed for quantitative antioxidant activity, 17 (54.8%) had activities higher than that of L-ascorbic acid (vitamin C). The higher antioxidant activities of the plant extracts is be due to the presence of more than 11 different antioxidant compounds present in some of the crude extracts (Fig. 1).

**Table 2:** Total phenolic content and DPPH free radical scavenging activity (EC_50_ (μg/ml)) of 31 methanol plant extracts of 31 different plant species

Plant species #	Total phenolics mg GAE/ g of extract	Antioxidant activity EC_50_ (μg/ml)	Percentage extract yield g/100 g dry material
1	8.56 ± 1.75	2.48 ± 1.11	21.68
2	7.66 ± 0.88	1.94 ± 0.44	22.10
3	7.43 ± 0.46	1.97 ± 0.24	26.89
4	7.03 ± 1.21	3.88 ± 0.64	30.15
5	9.21 ± 0.233	1.81 ± 0.40	30.62
6	14.58 ± 4.09	1.52 ± 0.30	13.86
7	11.40 ± 1.67	1.87 ± 0.08	26.37
8	8.75 ± 0.81	1.20 ± 0.22	13.67
9	10.61 ± 3.07	1.76 ± 0.28	17.35
10	10.39 ± 0.74	1.93 ± 0.86	9.33
11	9.43 ± 0.95	2.81 ± 1.10	17.14
12	6.53 ± 0.59	8.36 ± 1.37	16.41
13	8.71 ± 1.34	1.61 ± 0.27	14.20
14	8.32 ± 3.03	3.88 ± 0.64	23.95
15	13.61 ± 7.47	1.52 ± 0.59	15.10
16	17.66 ± 3.00	1.30 ± 0.10	23.55
17	8.73 ± 2.80	1.54 ± 0.52	36.60
18	18.54 ± 1.43	1.58 ± 0.54	29.53
19	8.51 ± 3.30	4.02 ± 0.43	26.64
20	8.40 ± 1.12	8.18 ± 0.72	32.14
21	16.35 ± 1.97	1.62 ± 0.21	25.65
22	6.73 ± 1.86	8.69 ± 0.03	28.16
23	8.04 ± 2.64	3.38 ± 0.08	16.04
24	7.93 ± 1.26	8.23 ± 0.84	30.02
25	18.65 ± 3.86	4.20 ± 3.39	20.42
26	16.08 ± 1.93	1.62 ± 0.01	25.18
27	15.60 ± 2.06	1.45 ± 0.64	35.49
28	10.32 ± 4.24	1.48 ± 0.30	43.45
29	7.64 ± 0.25	3.25 ± 2.15	37.85
30	12.35 ± 0.40	12.14 ± 1.11	24.19
31	5.17 ± 0.97	19.07 ± 1.50	22.10
Ascorbic acid	-	2.28 ± 0.02	

**Fig. 2:**
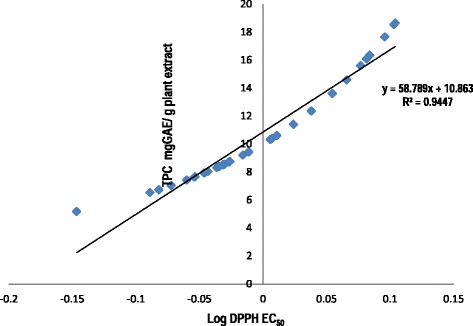
Correlation between antioxidant activity and total phenolic content of methanol extracts of the 31 selected plant species

Polyphenolic compounds may constitute the main class of natural antioxidants present in plants, food and beverages [26]. The total phenolic content of 31 extracts ranged from 5.17 ± 0.97 to 18.65 ± 3.86 mgGAE/g plant extract. The total phenolic content of the plant extracts correlated well with the respective antioxidative activity of the plant extracts (Fig. 2). Good correlation was found between the gallic acid equivalent/mg and the logarithm of EC_50_ values (R^2^˃ 0.9447). Phenolic constituents react with active oxygen radicals such as hydroxyl radical, superoxide anion radical and lipid peroxyl radical [27, 28]. These compounds have a broad spectrum of chemical and biological activities including radical scavenging properties.

The results for the mutagenic effects of 31 plant leaf extracts in the Ames test (*S. typhimurium* TA98 and TA100) are summarised in Table 3. The tester strains used in this study were selected because they are sensitive and detect a large proportion of known bacterial mutagens and are most commonly used routinely within the pharmaceutical industry [29]. Only one plant extract; *Halleria lucida* (#3) was mutagenic in TA98 (Table 3). The mutagenicity of *Halleria lucida* may be due to histidine and histidine related precursors present in plant extracts which often interfere with the Ames test by increasing the number of spontaneous revertants thus resulting in false-positive results [30]. In some cases, the number of colonies in test sample (eg. sample 21) is lower than the negative control. For all the extracts tested in the current experiments, the density of background bacterial lawn was compared to that of the negative control (after 48 h) and had no visible differences, indicating a lack of toxicity to the bacteria at the concentration tested. It is a known phenomenon that higher plants often produce antimicrobial agents and these can kill the tester strain [31]. Although the bacterial lawn was present, the low numbers of revertant colonies in *S. typhimurium* TA100 may indicate toxicity since the tester strain TA100 is more sensitive to toxic substances than strain TA98 [32]. An alternative explanation could be that the plant extracts induced a mild stress resulting in activation of DNA repair mechanisms. This reduces the number of revertants even in ‘control’ cultures (where no DNA damage was induced). In other words, normally, spontaneous mutations occur in control cultures because there was some DNA damage that was not important enough to activate DNA repair mechanisms. Now, with the plant extracts there is (also) no induced DNA damage (control level of revertants/mutations) but DNA repair activation occurred, repairing the spontaneous mutations.

**Table 3:** Mean number of revertants per plate (± SD) in *S. typhimurium* TA 98 and TA 100 exposed to different concentrations of the 31 methanol plant plant extracts

Plant #/Conc.	*S. typhimurium* TA 98			*S. typhimurium* TA 100		
	5 mg/ml (500 μg/plate)	0.5 mg/ml (50 μg/plate)	0.05 mg/ml (5 μg/plate)	5 mg/ml (500 μg/plate)	0.5 mg/ml (50 μg/plate)	0.05 mg/m (5 μg/plate)
1	33.33 ± 5.03	36.33 ± 4.04	43.33 ± 5.51	134.00 ± 16.09	139.00 ± 18.52	135.33 ± 7.63
2	25.33 ± 4.50	42.33 ± 7.30	36.00 ± 6.12	151.00 ± 16.64	139.67 ± 7.50	136.67 ± 9.86
3	53.67 ± 3.51	63.33 ± 4.93	58.33 ± 6.35	124.33 ± 6.43	137.67 ± 4.93	155.00 ± 3.60
4	23.00 ± 6.24	20.00 ± 4.04	24.00 ± 5.20	137.67 ± 11.59	143.00 ± 5.57	143.67 ± 2.52
5	28.67 ± 7.10	16.90 ± 5.69	18.67 ± 3.78	135.33 ± 5.13	117.67 ± 6.80	127.67 ± 15.95
6	23.67 ± 2.52	23.33 ± 3.05	19.00 ± 1.00	109.33 ± 10.12	127.33 ± 4.93	145.00 ± 3.51
7	32.33 ± 2.52	29.67 ± 7.02	19.67 ± 3.51	111.33 ± 10.07	137.00 ± 12.12	138.67 ± 11.72
8	34.67 ± 4.51	24.67 ± 7.23	21.33 ± 2.52	113.00 ± 3.61	134.00 ± 4.04	120.33 ± 4.04
9	29.33 ± 5.03	28.67 ± 5.51	25.67 ± 3.21	112.67 ± 2.04	126.00 ± 7.58	122.67 ± 11.37
10	27.00 ± 2.00	26.67 ± 1.53	26.67 ± 2.08	101.67 ± 5.13	119.67 ± 4.51	104.67 ± 5.69
11	28.00 ± 3.60	29.00 ± 6.24	25.67 ± 4.51	101.33 ± 4.16	90.00 ± 3.61	93.83 ± 2.52
12	22.33 ± 6.81	23.67 ± 4.04	25.67 ± 3.05	101.33 ± 1.53	115.00 ± 12.06	127.33 ± 3.21
13	19.33 ± 6.35	24.67 ± 6.43	19.00 ± 3.61	109.33 ± 9.71	104.00 ± 5.00	116.00 ± 4.58
14	15.00 ± 5.29	29.00 ± 9.54	29.33 ± 5.69	100.67 ± 4.16	101.67 ± 7.37	96.67 ± 9.71
15	22.67 ± 1.15	28.33 ± 7.50	23.67 ± 1.53	92.67 ± 6.43	96.33 ± 3.21	99.33 ± 3.06
16	19.67 ± 4.16	22.00 ± 2.64	20.67 ± 4.16	91.67 ± 3.21	85.00 ± 3.00	90.33 ± 2.31
17	24.33 ± 4.51	25.00 ± 4.00	22.67 ± 5.13	104.00 ± 5.29	91.00 ± 4.60	98.00 ± 2.00
18	24.00 ± 2.00	21.67 ± 2.08	26.67 ± 6.03	95.67 ± 2.52	82.67 ± 3.79	96.67 ± 1.53
19	26.00 ± 5.57	23.00 ± 6.00	25.33 ± 8.14	89.33 ± 10.69	81.33 ± 1.52	102.33 ± 2.08
20	28.67 ± 6.11	24.33 ± 1.15	21.33 ± 3.05	70.00 ± 7.55	84.33 ± 1.53	74.00 ± 7.80
21	23.33 ± 6.03	21.33 ± 2.08	25.00 ± 2.64	49.33 3.79	84.00 ± 10.15	77.00 ± 8.08
22	16.00 ± 1.00	19.67 ± 3.05	18.67 ± 5.51	87.67 ± 8.62	83.33 ± 4.73	86.33 ± 3.3.22
23	18.00 ± 4.36	19.67 ± 4.04	20.33 ± 1.53	88.00 ± 1.73	88.33 ± 14.50	89.00 ± 3.06
24	21.33 ± 3.21	25.00 ± 6.24	19.33 ± 3.05	82.33 ± 7.37	83.33 ± 2.89	86.00 ± 2.65
25	40.00 ± 3.60	35.00 ± 8.54	35.33 ± 2.89	102.00 ± 7.94	127.33 ± 10.69	119.00 ± 11.27
26	38.67 ± 11.06	33.33 ± 9.50	34.33 ± 6.66	114.00 ± 14.00	104.00 ± 6.00	92.50 ± 7.00
27	36.67 ± 8.14	42.67 ± 2.08	36.66 ± 2.08	122.67 ± 11.59	107.33 ± 6.03	124.00 ± 4.58
28	37.00 ± 2.64	36.67 ± 6.02	34.67 ± 4.51	119.00 ± 6.00	94.00 ± 4.16	104.00 ± 8.50
29	28.33 ± 9.71	29.00 ± 6.00	33.00 ± 9.16	97.50 ± 5.13	86.50 ± 5.69	100.00 ± 8.72
30	33.33 ± 6.11	34.67 ± 4.04	29.67 ± 6.81	98.67 ± 5.03	91.66 ± 9.45	98.67 ± 5.51
31	25.33 ± 4.04	35.00 ± 3.60	33.00 ± 11.00	82.67 ± 2.52	104.67 ± 5.51	104.00 ± 9.64
Solvent blank	24.7 ± 6.59				119.90 ± 9.85	
4NQO	239.44 ± 17.31				1082.34 ± 63.91	

The background levels as well as positive control values were in all cases within the normal limits found in our laboratory and in accordance with literature data [33]. The absence of mutagenic response by plant extracts against *Salmonella typhimurium* bacterial strains in the Ames test is a positive step in the safe use of plants in traditional medicine [34]. An extensive database has shown that many chemicals that are positive in the Ames test also have mutagenic activity in other tests [20]. Moreover, the proportion of carcinogens identified as mutagens by the Ames test ranges from about 50 to 90% [33].

**Fig. 3:**
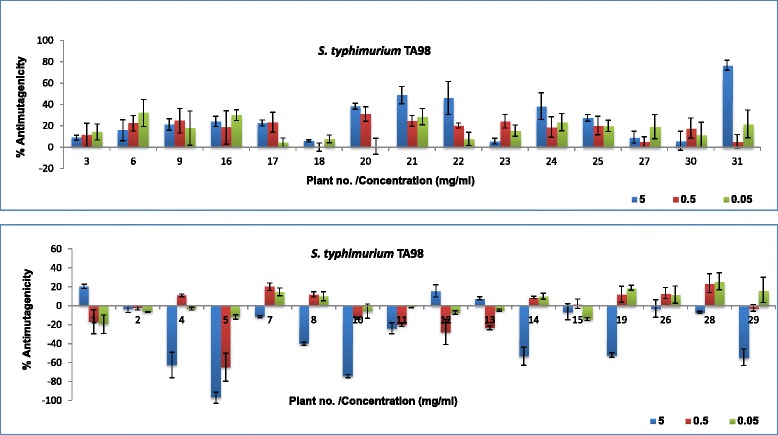
Percentage inhibition of mutagenic effects of 4-NQO by 31 methanol plant extracts in the Ames test using *S. typhimurium* TA98

**Fig. 4:**
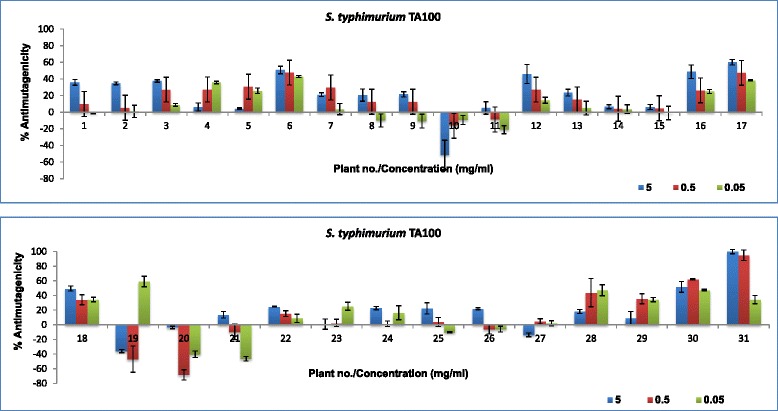
Percentage inhibition of mutagenic effects of 4-NQO by 31 methanol plant extracts in the Ames test using *S. typhimurium* TA100. Plant numbers refer to plant species examined Table 1

To determine the potential antimutagenic activity of the plant extracts to prevent DNA damage by 4-NQO (positive mutagen/carcinogen), plant extracts were incubated together with 4-NQO. The results are presented in Fig. 3 and Table 4. The percentage inhibition of mutagenic activity of 4-NQO (antimutagenicity) of the plant extracts ranged from 0.8 to 77% in *S. typhimurium* TA98 (Fig. 3) and from 0.8 to 100% in *S. typhimurium* TA100 (Fig. 4). In *S. typhimurium* TA98, eight extracts had more than 25% antimutagenic activity. Only three extracts had 45% and more antimutagenic activity even at the highest concentration tested. in the *S. typhimurium* TA100 assay, nine plant extracts had more than 25% antimutagenic activity and only seven extracts had 45% and more antimutagenic activity. In the Ames test, the antimutagenic effect is considered moderate when the inhibitory effect is between 25 − 40% and strong when more than 45%. Inhibitory effects of less than 25% are considered weak [35, 36]. Based on the conclusion of Negi and colleagues [35], most of the plant extracts assayed in this study may be considered to have weak or no antimutagenic effects. However, other authors consider a 20% decrease in the number of revertants to score the extract as active [31].

**Table 4:** Antimutagenic effects of 31 methanol plant extracts in *S. typhimurium* TA98 and TA100 tester strains

Sample # Conc.(mg/ml)	TA 98			TA 100		
	5	0.5	0.05	5	0.5	0.05
1	180.00 ± 34.59 [20.68 ± 2.27]	253.67 ± 16.65 [−16.44 ± 1.62]	260.67 ± 15.04 [−19.99 ± 3.68]	662.67 ± 8.09 [39.09 ± 3.14]	877.33 ± 15.18 [14.28 ± 2.58]	1001.33 ± 6.43 [−0.21 ± 0.78]
2	227.10 ± 24.31 [-3.22±2.44]	226.00 ± 11.68 [−2.71 ± 2.23]	233.67 ± 10.01 [−6.27 ± 0.37]	673.67 ± 13.87 [37.79 ± 1.68]	961.67 ± 4.58 [4.54 ± 0.56]	1000.67 ± 20.74 [0.08 ± 1.38]
3	20.4.67±24.09 [8.48±2.39]	199.67 ± 16.86 [11.02 ± 1.06]	190.33 ± 18.15 [15.60 ± 3.47]	657.00 ± 11.36 [39.67 ± 1.38]	760.67 ± 12.67 [29.40 ± 1.54]	940.67 ± 12.50 [6.73 ± 1.54]
4	343.00±32.69 [-62.26±13.47]	200.67 ± 36.35 [10.52 ± 1.49]	227.67 ± 35.81 [−3.22 ± 1.47]	948.67 ± 29.40 [4.66 ± 4.82]	802.00 ± 24.64 [18.01 ± 2.40]	690.00 ± 16.09 32.43 ± 1.57]
5	417.67±14.19 [-97.89±5.84]	347.67 ± 36.07 [−64.30 ± 14.86]	243.00 ± 5.57 [−11.36 ± 2.29]	955.00 ± 10.15 [9.89 ± 0.99]	760.00 ± 26.63 [38.88 ± 2.60]	803.55 ± 34.075 [24.99 ± 3.32]
6	190.67±24.09 [15.61 ± 9.92]	177.67 ± 17.78 [22.23 ± 7.32]	159.33 ± 30.83 [31.39 ± 12.70]	518.00 ± 26.51 [52.19 ± 4.53]	613.67 ± 17.52 [45.50 ± 1.71]	635.33 ± 12.66 [42.39 ± 1.23]
Solvent blank	24.2 ± 5.63			134.40 ± 3.53		
Positive control	220.67 ± 10.97			1001.00 ± 17.43		
7	230.67 ± 21.50 [−11.33 ± 1.08]	110.00 ± 7.94 [53.39 ± 4.00]	182.67 ± 8.33 [14.56 ± 4.20]	995.00 ± 29.87 [15.52 ± 2.35]	903.00 ± 14.53 [24.35 ± 11.42]	1116.67 ± 8.54 [3.79 ± 6.72]
8	285.33 ± 29.50 [−40.99 ± 1.49]	156.33 ± 16.44 [28.59 ± 2.84]	157.67 ± 9.45 [28.05 ± 4.78]	843.33 ± 9.45 [30.14 ± 7.43]	733.33 ± 12.67 [40.74 ± 9.95]	826.67 ± 10.02 [31.75 ± 7.87]
9	170.67 ± 10.69 [2.03 ± 5.39]	164.67 ± 22.68 [24.27 ± 11.40]	176.33 ± 31.66 [17.80 ± 5.79]	853.33 ± 4.04 [29.17 ± 3.18]	1023.00 ± 11.36 [12.82 ± 8.93]	1262.67 ± 10.50 [−10.28 ± 8.26]
10	347.67 ± 20.21 [−74.43 ± 1.76]	232.67 ± 16.26 [−12.40 ± 1.17]	219.67 ± 10.50 [5.39 ± 7.57]	1686.67 ± 9.74 [−51.15 ± 17.75]	1330.00 ± 18.77 [−16.77 ± 6.70]	1253.33 ± 15.14 [−9.38 ± 5.40]
11	252.67 ± 8.14 [−23.19 ± 5.87]	247.67 ± 23.75 [−20.49 ± 1.71]	210.00 ± 14.80 [−0.92 ± 1.07]	1110.00 ± 21.00 [4.43 ± 7.49]	1243.33 ± 4.04 [−8342 ± 1.44]	1377.33 ± 13.11 [−21.33 ± 4.68]
12	180.00 ± 8.88 [15.64 ± 6.41]	230.00 ± 18.52 [−27.51 ± 13.35]	223.67 ± 26.10 [−7.55 ± 1.88]	681.40 ± 15.03 [45.75 ± 11.43]	873.33 ± 3.05 [27.25 ± 1.09]	1006.67 ± 10.21 [14.39 ± 3.64]
Solvent blank	23.60 ± 2.06			118.06 ± 3.21		
Positive control	209.00 ± 2.00			1156.80 ± 17.43		
13	209.00 ± 19.00 [7.68 ± 1.73]	273.00 ± 24.06 [−23.71 ± 2.17]	234.67 ± 13.27 [−4.58 ± 1.21]	976.00 ± 13.05 [26.66 ± 3.97]	1108.33 ± 2.00 [15.54 ± 0.61]	1243.67 ± 27.30 [4.17 ± 8.31]
14	333.00 ± 10.44 [−53.14 ± 9.51]	207.00 ± 13.00 [8.67 ± 1.81]	156.00 ± 15.17 [33.68 ± 3.21]	1242.00 ± 7.94 [4.31 ± 2.42]	1216.67 ± 2.11 [6.44 ± 6.73]	1250.00 ± 16.77 [3.64 ± 5.11]
15	238.67 ± 1.15 [−6.54 ± 2.47]	219.33 ± 5.50 [2.39 ± 1.02]	252.00 ± 17.69 [−13.40 ± 1.61]	1220.00 ± 10.26 [6.16 ± 3.13]	1236.00 ± 8.00 [4.82 ± 2.44]	1313.33 ± 26.51 [−1.68 ± 2.07]
16	175.33 ± 8.73 [24.36 ± 4.71]	187.67 ± 29.14 18.48 ± 5.72]	163.33 ± 9.29 [30.25 ± 5.01]	719.67 ± 5.04 [48.19 ± 8.21]	976.33 ± 13.57 [26.63 ± 3.18]	1209.67 ± 10.44 [7.03 ± 2.45]
17	179.00 ± 5.20 [22.40 ± 2.80]	177.00 ± 17.35 [23.38 ± 9.36]	216.33 ± 8.08 [4.25 ± 2.36]	576.00 ± 14.01 [60.26 ± 3.28]	723.33 ± 5.29 [47.88 ± 0.35]	836.67 ± 3.21 [38.36 ± 0.75]
18	220.00 ± 2.00 [2.29 ± 1.08]	224.00 ± 7.00 [0.33 ± 3.78]	210.67 ± 6.81 [7.19 ± 3.67]	700.00 ± 14.73 [49.84 ± 3.45]	886.67 ± 9.59 [34.16 ± 6.94]	880.00 ± 13.43 [34.72 ± 3.14]
Solvent blank	20.8 ± 4.18			103.00 ± 3.74		
Positive control	224.67 ± 9.45			1293.33 ± 8.08		
19	322.33 ± 49.52 [−51.77 ± 2.48]	195.50 ± 16.65 [12.33 ± 8.35]	182.67 ± 5.13 [19.06 ± 2.57]	1350.00 ± 30.00 [−35.35 ± 2.24]	1450.33 ± 10.27 [−46.23 ± 17.88]	1570.0 ± 26.46 [−59.28 ± 7.01]
20	144.33 ± 5.69 [38.28 ± 2.28]	159.00 ± 13.53 [30.69 ± 6.78]	226.00 ± 15.13 [−3.20 ± 7.59]	1056.67 ± 15.27 [−3.44 ± 1.68]	1653.33 ± 35.12 [−68.35 ± 7.02]	1396.67 ± 20.82 [−40.43 ± 4.32]
21	123.33 ± 16.04 [48.65 ± 8.04]	171.67 ± 10.02 [24.62 ± 5.02]	164.67 ± 15.01 [28.16 ± 7.52]	903.33 ± 5.77 [13.23 ± 4.51]	1112.67 ± 11.01 [−9.53 ± 10.40]	600.00 ± 34.64 [46.23 ± 3.016]
22	130.67 ± 26.01 [45.36 ± 15.46]	180.00 ± 4.00 [20.07 ± 2.37]	205.00 ± 10.44 [7.42 ± 6.21]	590.00 ± 5.77 [24.84 ± 0.59]	880.00 ± 26.46 [15.77 ± 4.03]	943.33 ± 11.55 [8.88 ± 5.62]
23	209.67 ± 4.51 [5.40 ± 2.68]	172.33 ± 10.69 [24.12 ± 6.36]	190.00 ± 8.72 [15.01 ± 5.18]	1017.67 ± 5.01 [0.80 ± 1.95]	1005.67 ± 10.69 [2.10 ± 4.91]	710.00 ± 10.16 [25.56 ± 5.60]
24	144.33 ± 21.13 [38.28 ± 12.56]	183.33 ± 16.17 [18.30 ± 9.61]	173.67 ± 13.65 [23.61 ± 8.12]	816.67 ± 22.66 [22.66 ± 2.12]	1011.33 ± 12.05 [1.49 ± 3.86]	870.00 ± 26.46 [16.86 ± 9..48]
Solvent blank	22.00 ± 4.61			105.67 ± 5.59		
Positive control	219.67 ± 9.89			1025.60 ± 35.79		
25	1607.67 ± 4.93 [27.89 ± 3.20]	174.67 ± 13.32 [20.24 ± 8.69]	186.33 ± 8.02 [13.68 ± 5.21]	856.67 ± 15.27 [22.76 ± 7.68]	1040.00 ± 10.00 [3.76 ± 6.13]	1180.00 ± 20.00 [−10.73 ± 0.63]
26	217.67 ± 14.29 [−3.28 ± 9.28]	186.33 ± 8.96 [13.68 ± 8.82]	190.00 ± 14.00 [11.49 ± 9.09]	870.00 ± 20.00 [21.38 ± 1.25]	1126.67 ± 30.55 [−5.22 ± 6.45]	1132.00 ± 25.53 [−5.77 ± 3.90]
27	194.67 ± 6.66 [9.30 ± 5.44]	202.33 ± 8.39 [4.92 ± 4.87]	175.33 ± 7.50 [19.69 ± 8.34]	1203.33 ± 26.17 [−13.16 ± 2.66]	1035.00 ± 5.00 [4.28 ± 3.74]	1057.33 ± 11.59 [1.97 ± 1.42]
28	223.33 ± 17.01 [−6.56 ± 1.13]	168.33 ± 14.84 [23.52 ± 9.89]	141.33 ± 13.58 [25.16 ± 9.09]	911.00 ± 20.05 [17.13 ± 2.75]	749.67 ± 11.93 [33.85 ± 9.43]	580.00 ± 17.32 [51.44 ± 7.54]
29	261.67 ± 13.05 [−54.16 ± 8.70]	165.67 ± 5.13 [−2.19 ± 3.42]	155.00 ± 19.97 [16.95 ± 3.32]	1031.67 ± 10.41 [4.63 ± 4.31]	760.00 ± 20.00 [32.78 ± 6.84]	746.67 ± 15.27 [34.16 ± 2.87]
30	200.00 ± 13.23 [6.01 ± 8.82]	179.50 ± 14.19 [17.23 ± 9.46]	190.67 ± 17.47 [11.49 ± 1.64]	583.33 ± 20.82 [51.09 ± 7.23]	476.67 ± 15.27 [62.14 ± 0.66]	615.00 ± 35.00 [47.81 ± 1.16]
31	71.00 ± 7.00 [76.58 ± 4.67]	201.67 ± 9.45 [5.47 ± 6.30]	171.33 ± 19.65 [21.88 ± 13.1]	118.00 ± 15.62 [99.32 ± 2.95]	160.00 ± 10.00 [94.96 ± 7.25]	746.67 ± 45.09 [34.16 ± 5.59]
Solvent blank	28.2 ± 6.57			111.40 ± 11.13		
Positive control	211.67 ± 7.57			1076.33 ± 26.95		

The results are expressed as mean number of revertants/plate (± SD) percentage antimutagenicity [in squared brackets]

It appears the tested plant extracts have more antimutagenic activity in the *S. typhimurium* TA100 than in *S. typhimurium* TA98 assay which may be related the mode and/or mechanism of the antimutagenic effects. Almost 50% of the plant extracts reduced the mutagenic effects of 4-NQO in *S. typhimurium* TA98 and 77% in *S. typhimurium* TA100. The remaining plants increased the mutagenic effects of the mutagen 4-NQO, a phenomenon known as co-mutagenicity. A co-mutagenic effect is observed in instances where, when tested alone, plant extracts don’t have any mutagenic effects but in the presence of a positive mutagen, these extracts enhance or increase the mutagenicity of the positive control.

Not all antimutagenic plant extracts had activity in both *S. typhimurium* TA98 and *S. typhimurium* TA100. It is possible that these extracts have multiple mechanisms of mutation inhibition/antimutagenesis since they prevent frame-shift mutations detectable in TA98 and base-pair substitutions detectable in TA100. This is one of the many advantages of using the Ames test in antimutagenesis studies as it provides information not only of antimutagenesis but also on possible mode of action (De Flora et al., [37]).

Only extracts of ten plant species had antimutagenic activity in both strains. These extracts may have potential antimutagenic compounds because one of the most important characteristics of antimutagens is their universality, in this case being the inhibition of mutations resulting from both reverse frame shift or base-pair substitutions [35]. The differences in the observed activities in the other plant extracts may be due to compounds contained within the extracts that can only prevent, inhibit and/or reverse frame shift or base-pair substitutions.

Plant extracts with good antimutagenic effects in *S. typhimurium* TA98 in general had good antioxidant activity and relatively higher phenolic content. Plant extracts with good antimutagenic effects in *S. typhimurium* TA100 generally had higher antioxidant activity but with lower phenolic content. It is clear that there is a direct correlation between antioxidant activity and antimutagenicity. There is also a direct correlation between antioxidant activity and total phenolic contents of the extracts tested in this study. The relationship between antioxidant activity and total phenolics in all plant extracts is in agreement with the results of other authors. Good correlation was found between the mg GAE/g and the logarithm of EC_50_ values (R^2^˃ 0.9447). Polyphenols have been reported to be responsible for the antioxidant activity in plant extracts [23]. Phenolic constituents react with active oxygen radicals such as hydroxyl radical, superoxide anion radical and lipid peroxyl radical [25]. In addition to radical scavenging properties these compounds have a broad spectrum of chemical and biological activities.

## Conclusions

Some of the plant extracts investigated have potential antimutagenic activity as evident in the Ames test using *S. typhimurium* TA98 and TA100. Antioxidant compounds in the plant extracts appear to be responsible for their antimutagenic activity. There is a clear correlation between antioxidant activity and antimutagenicity of the extracts tested in this study. We assume that it is the presence of antioxidant polyphenols acting against 4-NQO that decreases the incidence of DNA mutation. Some of the species examined here contained at least 11 different compounds with antioxidant activity.

It is so much easier to determine antioxidant activity and the number of antioxidant compounds present in extracts than carrying out antimutagenic assays that this approach could be a first step in searching for extracts with compounds that can be used against cancer. Variations in the antioxidant activities of the plant extracts may be largely attributed to differences in the quality and quantity of phenolic compounds and other bioactive compounds present in the extracts. Investigation of extracts of plants that are not mutagenic, cytotoxic and that have antimutagenic activity, may lead to identification of compounds that can prevent broad spectrum human diseases caused by mutations. Based on these findings, several plant extracts that have the desired effects will be further investigated for their antigenotoxic effects in the cytokinesis-block micronucleus/cytome assay and single cell gel electrophoresis/comet assay to further establish their potential to prevent chromosomal aberrations and DNA fragmentation.

## Additional file

Additional file 1: List of 120 plant species that were extracted with methanol to determine quantitative antioxidant activity in order to select 31 plant species for further work. (DOCX 38 kb)

## Abbreviations

4-NQO: 4-nitroquinoline 1-oxide
DNA: Deoxyribonucleic acid
DPPH: 2, 2-diphenyl-1-picrylhydrazyl
EC_50_: Effective concentration
GAE: Gallic acid equivalents
RNS: Reactive nitrogen species
ROS: Reactive oxygen species
TLC: Thin layer chromotography
UV: Ultra violet
