# Supplementary material for: The correlation between antimutagenic activity and total phenolic content of extracts of 31 plant species with high antioxidant activity
Source: BMC Complement Altern Med. 2016 Nov 29;16:490. doi: 10.1186/s12906-016-1437-x (PMC5129238; doi:10.1186/s12906-016-1437-x)
Supplement: Additional file 1: — List of 120 plant species that were extracted with methanol to determine quantitative antioxidant activity in order to select 31 plant species for further work. (DOCX 38 kb) [file 12906_2016_1437_MOESM1_ESM.docx]

SUPPLEMENTARY INFORMATION

**List of Plant species tested for qualitative antioxidant activity using a combination of thin layer chromatography (TLC) and 2, 2-diphenyl-1-picrylhydrazyl (DPPH)**

| **PMDN Number** | **Genus and species name** | **Family** | |
| --- | --- | --- | --- |
| 65 | *Pappea capensis* Eckl. & Zeyh. | | Sapindaceae |
| 69 | *Rhus leptodictya* Diels | | Anacardiaceae |
| 95 | *Zanha golungensis* Hiern | | Sapindaceae |
| 102 | *Berchemia discolor* (Klotzsch) Hemsl. | | Rhamnaceae |
| 106 | *Combretum nelsonii* Dummer | | Combretaceae |
| 107 | *Commiphora harveyi* (Engl.) Engl. | | Burseraceae |
| 111 | *Dolichandrone alba* (Sim) Sprague | | Bignoniaceae |
| 121 | *Hymenodictyon parvifolium* Oliv. | | Rubiaceae |
| 130 | *Sclerocarya birrea*  (A. Rich.) Hochst | | Anacardiaceae |
| 363 | *Acalypha glabrata* Thunb. | | Euphorbiaceae |
| 364 | *Azanza garckeana* (F.Hoffm.) Exell & Hillc. | | Malvaceae |
| 365 | *Catunaregam spinosa* (Thunb.) Tirveng | | Rubiaceae |
| 367 | *Dalbergia nitidula* Baker | | Leguminosae |
| 370 | *Freylinia lanceolata* (L.) G. Don | | Scrophulariaceae |
| 371 | *Gymnosporia nemorosa* (Eckl. & Zeyh.) Szyszył | | Celastraceae |
| 372 | *Halleria lucida* L. | | Stilbaceae |
| 373 | *Karomia speciose* (Hutch & Corbishley) R.Fern. | | Lamiaceae |
| 374 | *Maerua rosmarinoides* Gilg & Gilg-Ben. | | Capparaceae |
| 375 | *Mystroxylon aethiopicum* (Thunb.) Loes. | | Celastraceae |
| 376 | *Pancovia golungensis* (Hiern) Excell & Mendonça | | Sapindaceae |
| 378 | *Pseudosalacia streyi* Codd | | Celastraceae |
| 379 | *Pterocelastrus echinatus* N.E. Br. | | Celastraceae |
| 380 | *Putterlickia retrospinosa* A.E. van Wyk Mostert | | Celastraceae |
| 381 | *Putterlickia pyracantha* (L.) Endl. | | Celastraceae |
| 383 | *Tannodia swynnertonii* (S. Moore) Prain | | Euphorbiaceae |
| 384 | *Thespesia acutiloba* (Baker f.) Excell & Mendonça | | Malvaceae |
| 385 | *Triplochiton zambesiacus* Milne-Redh. | | Malvaceae |
| 386 | *Turraea floribunda* Hochst. | | Meliaceae |
| 387 | *Uvaria gracillipes* N.Robson | | Annonaceae |
| 390 | *Acridocarpus natalitus* A.Juss. | | Malpighiaceae |
| 391 | *Alchornea hirtella* Benth. | | Euphorbiaceae |
| 392 | *Alchornea laxiflora* (Benth.) Pax & K.Hoffm. | | Euphorbiaceae |
| **PMDN Number** | **Genus and species name** | | **Family** |
| 393 | *Androstachys johnsonii* Prain | | Picrodendraceae |
| 394 | *Antidesma venosum* E.Mey. ex Tul. | | Phyllanthaceae |
| 395 | *Aporrhiza nitida* Gilg ex Milne-Redh. | | Sapindaceae |
| 396 | *Argomuellera macrophylla* Pax | | Euphorbiaceae |
| 397 | *Baphia racemosa* (Hochst.) Baker | | Leguminosae |
| 398 | *Barringtonia racemosa* (L.) Spreng. | | Lecythidaceae |
| 399 | *Brachystegia spiciformis* Benth. | | Leguminosae |
| 401 | *Cassinopsis tinifolia* Harv. | | Icacinaceae |
| 402 | *Chrysophyllum gorungosanum* Engl. | | Sapotaceae |
| 403 | *Clerodendrum glabrum* E.Mey. var. glabrum | | Lamiaceae |
| 404 | *Combretum bracteosum* (Hochst.) Engl. & Diels | | Combretaceae |
| 405 | *Combretum celastroides* Welw. ex M.A.Lawson | | Combretaceae |
| 406 | *Combretum collinum* subsp. *Suluense* (Engl. & Diels) Okafa | | Combretaceae |
| 407 | *Combretum microphyllum* Klotzsch | | Combretaceae |
| 410 | *Deinbollia oblongifolia* (E,Mey. ex Arn.) Radlk. | | Sapindaceae |
| 549 | *Synsepalum brevipes* (Baket) T.D. Penn. | | Sapotaceae |
| 577 | *Acokanthera oblongifolia* (Hochst.) Benth. & Hook.f. ex B.D. Jacks | | Apocynaceae |
| 582 | *Brabejum stellatifolium.* L | | Proteaceae |
| 583 | *Brachylaena discolor* DC. | | Compositae |
| 584 | *Buddleja salviifolia* (L.) Lam. | | Scrophulariaceae |
| 585 | *Burchellia bubalina* (L.f.) Sims | | Rubiaceae |
| 586 | *Cassine transvaalensis* (Burtt Davy) Codd | | Celastraceae |
| 587 | *Cassinopsis illicifolia* (Hochst.) Sleumer | | Icacinaceae |
| 588 | *Chaetachme aristata* Planch. | | Ulmaceae |
| 589 | *Chrysanthemoides molifera* (L.) Norl. | | Compositae |
| 591 | *Cordia caffra* Sond. | | Boraginaceae |
| 592 | *Croton gratissimus* Burch. | | Euphorbiaceae |
| 593 | *Croton sylvaticus* (Hochst.) | | Euphorbiaceae |
| 597 | *Duvernoia adhatodoides* E. Mey ex Nees | | Acanthaceae |
| 598 | *Ekebergia capensis* Sparrm. | | Meliaceae |
| 600 | *Englerophytum magalismontanum* (Sond.) T.D. Penn. | | Sapotaceae |
| 602 | *Faurea rochetiana*  (A. Rich) Chiov. ex Pic. Serm. | | Proteaceae |
| 603 | *Gardenia cornuta* Hemsl. | | Rubiaceae |
| 604 | *Grewia flavescens* Juss. | | Malvaceae |
| 605 | *Harpephyllum caffrum* Bernh. | | Anacardiaceae |
| 606 | *Jatropha curcas* L. | | Euphorbiaceae |
| 608 | *Kirkia wilmsii* Engl. | | Kirkiaceae |
| **PMDN Number** | **Genus and species name** | | **Family** |
| 610 | *Leucospermum erubescens* Rourke | | Proteaceae |
| 611 | *Lippia javanica* (Burm.f.) Spreng. | | Verbenaceae |
| 612 | *Lonchocarpus nellsii* (Schinz) Heering & Grimme | | Leguminosae |
| 614 | *Loxostylis alata* A. Spreng. ex Rchb**.** | | Anacardiaceae |
| 616 | *Mackaya bella* Harv. | | Acanthaceae |
| 617 | *Peltophorum africanum* Sond. | | Fabaceae |
| 620 | *Podocarpus elongatus* **(**Aiton) L'Hér. ex Pers. | | Podocarpaceae |
| 621 | *Podocarpus henkelii* Stapf ex Dallim. & B.D.Jacks. | | Podocarpaceae |
| 622 | *Polygala myrtifolia* L. | | Polygalaceae |
| 623 | *Portulacaria afra*Jacq. | | Didiereaceae |
| 624 | *Protea rubropilosa* Beard | | Proteaceae |
| 625 | *Ptaeroxylon obliquum* (Thunb.) Radlk. | | Rutaceae |
| 626 | *Rapanea melanophloeos* (L.) Mez | | Primulaceae |
| 629 | *Schrebera alata* (Hochst.) Welw | | Oleaceae |
| 645 | *Aloe plicatilis* (L.) Mill. | | Xanthorrhoeaceae |
| 692 | *Ochna gamostigmata* Du Toit | | Ochnaceae |
| 718 | *Buxus natalensis* (Oliv.) Hutch. | | Buxaceae |
| 719 | *Morella serrata* (Lam.) Killick | | Myricaceae |
| 722 | *Pachypodium lamerai* Drake | | Apocynaceae |
| 723 | *Salix mucronata* Thunb. | | Salicaceae |
| 724 | *Steganotaenia araliaceae* Hochst. | | Apiaceae |
| 725 | *Abutilon sonneratianum.*(Cav.) Sweet | | Malvaceae |
| 730 | *Erythrophysa transvaalensis* Verd. | | Sapindaceae |
| 731 | *Ficus sycomorus* L. | | Moraceae |
| 732 | *Gomphostigma virgatum* (L.f.) Baill. | | Scrophulariaceae |
| 733 | *Grewia flava* DC. | | Malvaceae |
| 734 | *Heteromorpha arborescens* (Spreng.) Cham. & Schltdl. | | Apiaceae |
| 736 | *Loxostylis alata* A. Spreng. ex Rchb. | | Anacardiaceae |
| 737 | *Mussaenda erythrophylla* Schumach. & Thonn. | | Rubiaceae |
| 738 | *Noltea Africana* (L.) Rchb. f. | | Rhamnaceae |
| 739 | *Ochna serrulata* Walp. | | Ochnaceae |
| 740 | *Oncoba spinosa* Forssk. | | Salicaceae |
| 741 | *Quisqualis indica* L. | | Combretaceae |
| 743 | *Securidaca longepedunculata* Fresen. | | Polygalaceae |
| 746 | *Aloe plicatilis* (L.) Mill. | | Xanthorrhoeaceae |
| **PMDN Number** | **Genus and species name** | | **Family** |
| 747 | *Apodytes geldenhuysii* A.E. van Wyk & Potgieter | | Icacinaceae |
| 748 | *Berzelia lanuginosa* (L.) Brongn. | | Bruniaceae |
| 749 | *Erica brachialis* Salisb. | | Ericaceae |
| 750 | *Euryops tenuissimus* Less.*d* | | Compositae |
| 751 | *Heeria argentea* Meisn. | | Anacardiaceae |
| 753 | *Leucadendron coniferum* Meisn. | | Proteaceae |
| 754 | *Leucadendron discolor* E. Phillips & Hutch. | | Proteaceae |
| 757 | *Mimetes cucullatus* R. Br.*c* | | Proteaceae |
| 759 | *Podalyria calyptrata* (Retz.) Willd. | | Leguminosae |
| 759 | *Podalyria calyptrata* (Retz.) Willd. | | Leguminosae |
| 760 | *Protea mundii* Klotzsch | | Proteaceae |
| 761 | *Protea cynaroides* (L.) L. | | Proteaceae |
| 762 | *Protea neriifolia* R. Br. | | Proteaceae |
| 763 | *Protea nitida* Mill. | | Proteaceae |
| 764 | *Psoralea pinnata* L. | | Leguminosae |
|  | | | |
